# What Is Moderate to Vigorous Exercise Intensity?

**DOI:** 10.3389/fphys.2021.682233

**Published:** 2021-09-22

**Authors:** Brian R. MacIntosh, Juan M. Murias, Daniel A. Keir, Jamie M. Weir

**Affiliations:** ^1^Faculty of Kinesiology, University of Calgary, Calgary, AB, Canada; ^2^School of Kinesiology, Western University, London, ON, Canada

**Keywords:** exercise prescription, health benefits of exercise, exercise for health, lifestyle, physical activity

## Abstract

A variety of health benefits associated with physical activity depends upon the frequency, intensity, duration, and type of exercise. Intensity of exercise is the most elusive of these elements and yet has important implications for the health benefits and particularly cardiovascular outcomes elicited by regular physical activity. Authorities recommend that we obtain 150min of moderate to vigorous intensity physical activity (MVPA) each week. The current descriptions of moderate to vigorous intensity are not sufficient, and we wish to enhance understanding of MVPA by recognition of important boundaries that define these intensities. There are two key thresholds identified in incremental tests: ventilatory and lactate thresholds 1 and 2, which reflect boundaries related to individualized disturbance to homeostasis that are appropriate for prescribing exercise. VT2 and LT2 correspond with critical power/speed and respiratory compensation point. Moderate intensity physical activity approaches VT1 and LT1 and vigorous intensity physical activity is between the two thresholds (1 and 2). The common practice of prescribing exercise at a fixed metabolic rate (# of METs) or percentage of maximal heart rate or of maximal oxygen uptake (V̇O_2_max) does not acknowledge the individual variability of these metabolic boundaries. As training adaptations occur, these boundaries will change in absolute and relative terms. Reassessment is necessary to maintain regular exercise in the moderate to vigorous intensity domains. Future research should consider using these metabolic boundaries for exercise prescription, so we will gain a better understanding of the specific physical activity induced health benefits.

## Introduction

Moderate to vigorous intensity physical activity (MVPA) is commonly recommended for health benefits (Tremblay et al., 2011), yet the majority of the population does not engage in physical activity of sufficient intensity and volume (Warburton et al., 2007; Borgundvaag and Janssen, 2017) to obtain these health benefits. The WHO and the Government of the United States of America^1^ recognize the added benefit of exercising at a greater intensity to improve cardiorespiratory fitness (Ross et al., 2015) and to reduce risk of mortality and morbidity (Lee and Paffenbarger Jr., 2000; Wen et al., 2011). However, prescribing exercise at the recommended intensity requires a clear understanding of what moderate to vigorous physical activity is. A recent review of intensity of exercise by Jamnick et al. (2020) makes it clear that intensity needs to be individualized relative to specific boundaries, reflecting precise metabolic conditions above which physiological homeostasis is challenged. Here, our purpose is to provide a framework for understanding “moderate to vigorous” physical activity intensities and to recommend strategies for their individual identification for exercise prescription. To accomplish this, we will describe exercise intensity in the context of the metabolic responses to incremental exercise and constant intensity exercise. We will then relate the common ways that moderate to vigorous exercise is described and identify which of those is most useful for accurate exercise prescription. This information will be of value to those who prescribe exercise and in the design and interpretation of future research concerned with the associated health benefits of regular physical activity.

## Exercise and Physical Activity

Physical activity is defined as: “…any bodily movement produced by skeletal muscles that results in energy expenditure” (Caspersen et al., 1985). Exercise, is “physical activity that is planned, structured, and repetitive…” for the purpose of maintaining or improving physical fitness (Caspersen et al., 1985). It is recognized that in general, the human population does not engage in physical activity of sufficient intensity or duration to obtain health benefits (Troiano et al., 2008; Borgundvaag and Janssen, 2017). Recognizing these definitions, it is exercise that must be prescribed by health-care practitioners and intensity is an important part of this prescription.

### Exercise Intensity

In its simplest form, exercise intensity refers to the rate of metabolic energy demand during exercise. Exercise intensity can be expressed in absolute terms (e.g., oxygen uptake in liters per min, power output in watts, heart rate in beats per min, and speed of locomotion in meters per s or km per h) or in relative terms (i.e., relative to any of the following: body weight, maximal oxygen uptake, maximal heart rate, or heart rate reserve). However, it must be recognized that genetics, fitness status, comorbidities, and other factors combine to affect the ability to maintain homeostasis during acute bouts of exercise and that adaptations to regular exercise will be obtained only when sufficient disturbance to homeostasis has occurred (Shephard, 1968; Daussin et al., 2008). For this reason, prescription should be individualized based on the expected metabolic disturbance elicited by the exercise. The most common way to try to identify the intensity at which this disruption to physiological homeostasis occurs is with an incremental exercise test.

### Incremental Tests to Detect Boundary Conditions

Incremental exercise tests represent the most common approach to identify the boundary conditions and the exercise domains they demarcate. To understand these boundary conditions and identify an intensity of exercise as moderate or vigorous for an individual, it is important to understand how the body responds as exercise intensity progressively increases and to recognize the measurable physiological response features that signify disturbances to homeostasis. The intensity of exercise at which these disturbances to homeostasis are detected depends on the energy cost of the exercise and the individual ability to provide that energy and perform the exercise with minimal disturbance to homeostasis. Although several variables can be used to detect disturbance to homeostasis, the indices that are primarily used are blood lactate concentration ([La]_b_), an indicator of glycolysis providing the energy for exercise, and ventilation, a variable responsible for maintaining adequate gas exchange for aerobic metabolism.

**Figure 1:**
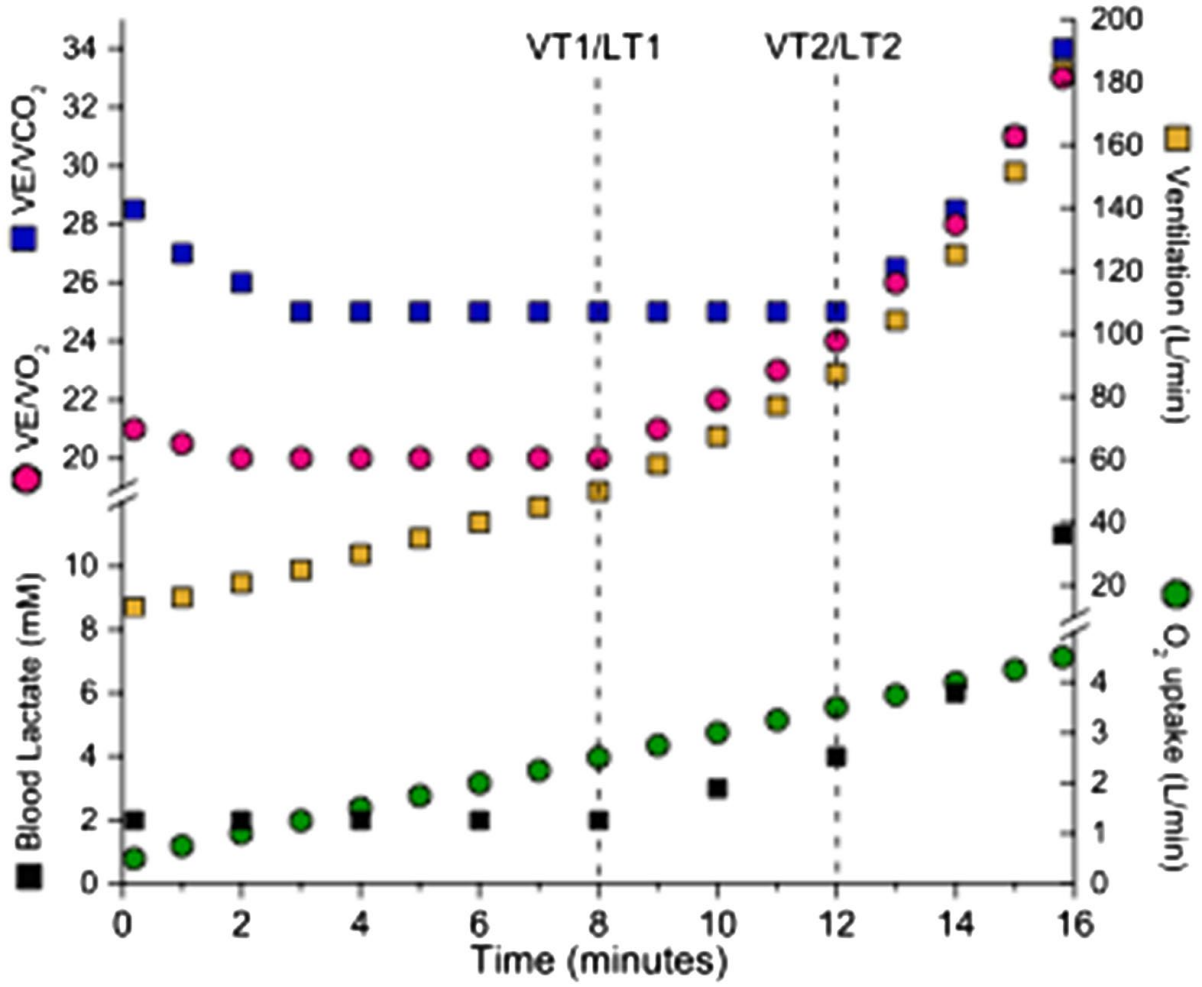
Incremental test for detection of thresholds. Pulmonary measurements and blood lactate concentration allow detection of boundary conditions known as first and second threshold (vertical dashed lines).

There are two primary boundary conditions, recognized as thresholds, and a variety of ways these can be detected during incremental tests. Observations during incremental tests are used to identify these thresholds. Intensity is usually quantified by oxygen uptake or power. Incremental tests can be step or ramp, but for power output to be relevant, step duration should be at least 2min and ramp slope should be slow. The first threshold represents the boundary between moderate and vigorous exercise and the second threshold represents the upper boundary of vigorous exercise. See Figure 1 for example.

The first threshold occurs at the metabolic rate associated with the initial challenge to physiological homeostasis. At the first threshold, ventilation increases relative to V̇O_2_ but remains proportional with V̇CO_2_. When detected this way, this threshold is referred to as the first ventilatory threshold (VT1) or gas exchange threshold. This first threshold is also detected as the first lactate threshold (LT1), corresponding to the intensity above which blood lactate rises noticeably above resting values (Beaver et al., 1985).

The second threshold represents the metabolic rate above which the disturbance to physiological homeostasis accelerates disproportionally. This is the upper boundary of vigorous exercise. Above the second threshold during an incremental test, ventilation increases disproportionately to V̇CO_2_ and end-tidal PCO_2_ falls from a prior period of stability. This second threshold is referred to as the second ventilatory threshold (VT2) or the respiratory compensation point when ventilatory parameters are used for detection. When this second threshold is detected by a substantial increase in blood lactate, it is called as the lactate threshold 2 (LT2). Several techniques are used to identify this increase in [La]_b_ (Svedahl and MacIntosh, 2003; Jules et al., 2018) including Dmax, a lactate threshold obtained by graphic technique that requires finding the longest perpendicular line from the line joining the lowest and highest [La]_b_ levels to the measured [La]_b_ (Płoszczyca et al., 2020). This second threshold can also be identified as the first intensity from which blood lactate changes by 1mM (Fletcher et al., 2010), or an inflection in a graph of [La]_b_ vs. intensity. Onset of blood lactate accumulation (OBLA), which corresponds to the lowest intensity of exercise during an incremental test that yields a blood lactate concentration of 4mM can also be used to detect LT2. The second threshold also corresponds to maximal lactate steady state (MLSS) and critical power or speed. These concepts are presented below.

The heart rate corresponding to these ventilatory or lactate thresholds can be used to prescribe exercise. There is a clear advantage of using an incremental test to identify these boundary conditions because both can be identified in a single test. Follow-up testing (reassessment) is necessary to monitor training-induced changes.

Incremental tests represent an effective way to identify these boundary conditions. Ramp tests are often used with reasonable success, as they accurately identify the oxygen uptake corresponding to these thresholds. However, the power output or treadmill speed at which the boundary is detected should not be used for exercise prescription due to the dissociation between V̇O_2_ and power output during ramp compared to constant-load exercise (Keir et al., 2018), unless very slow ramps (Iannetta et al., 2019) or a correction is introduced (Caen et al., 2020; Iannetta et al., 2020b). Step incremental tests, where each step is 2–3min in duration and beginning at least two intensities below the first threshold, are another useful alternative. In this case, the power output associated with the identified boundary is more likely to fall closer to that expected during constant-load exercise (although some level of uncertainty still remains). Incremental tests are also useful to identify a heart rate range or rating of perceived exertion associated with moderate and vigorous exercise.

### Homeostasis During Constant Intensity Exercise

There are two approaches using constant intensity trials that allow estimation of the second boundary conditions. The first is the MLSS and the second is the critical power/critical speed test. Both of these approaches yield intensities that closely approximate the metabolic rate (i.e., V̇O_2_) at the VT2 and LT2. The MLSS provides an estimate of the highest intensity for which a steady state oxygen uptake can account for all the energy cost of the exercise. Above this intensity there will be a sustained contribution from glycolysis leading to lactate accumulation in the blood. This test typically requires 2–5 trials with constant power output or constant speed, lasting 30min. MLSS is usually identified as the highest intensity of exercise with less than 1mM change in [La]_b_ between 10 and 30min, but smaller increments have been used (MacIntosh et al., 2002). This approach can be applied to several modes of locomotion such as, running, swimming, skating, and cross-country skiing. The disadvantages are that it may take several trials and that, inevitably, the true boundary condition will lie between two trials. This error can be diminished by using small increments of intensity between trials. Typically, trained individuals can sustain exercise at MLSS for 40–60min (Baron et al., 2008).

A second technique used to estimate the upper boundary condition with constant intensity trials is determination of critical power or critical speed. This technique requires multiple trials at an intensity above VT2. The endurance vs. distance (or work) relationship is plotted and fit with a straight line. The slope of the relationship is critical speed or critical power. This intensity of exercise corresponds well with the MLSS (Keir et al., 2015). Disadvantages of this measurement include: several trials are required and the trials require effort to exhaustion to accurately find a representation of this boundary condition. Additionally, unless a verification test is performed, the physiological responses at this critical intensity are not known.

### The Confusing Terminology

The problem associated with naming the boundary conditions is that there is a myriad of ways that these thresholds have been described and named (Svedahl and MacIntosh, 2003). For example, Bishop (Bishop et al., 1998) compared six different ways that lactate threshold can be identified. For this reason, it is important to pay attention to how a threshold is detected in any given research paper and recognize that the terminology may be defined differently in different papers. There are a multitude of thresholds identified with ramp and step incremental tests and prolonged trials to exhaustion. To make things more confusing, these terms are not used in a consistent manner. For example, the term “**anaerobic** threshold,” was first used to identify an intensity that aligns well with the VT1 but has also been used in a manner consistent with VT2 (Svedahl and MacIntosh, 2003) and lactate threshold detected by the Dmax method (Płoszczyca et al., 2020).

### Current Definitions of Moderate and Vigorous Exercise Intensity

Many organizations provide descriptions of moderate and vigorous exercise for the purpose of meeting physical activity guidelines. The information in Table 1 reflects the intensity description of three of these: the WHO, the United States Government and the Canadian Government. These descriptions of exercise intensity are summarized below and specific concerns are raised.

**Table 1:** Methods commonly used to prescribe moderate to vigorous intensity exercise.

Moderate intensity	Moderate intensity (description and/or examples provided)	Vigorous intensity	Vigorous intensity (description and/or examples provided)
3–<6 METs Walking 2.5–4 mph RPE=5–6 40–59% HRR	Walking briskly, dancing, and playing doubles tennis, or raking the yard, slow and swimming	>6 METs Fast walking >4mph RPE=7–8 60–84% HRR	Jogging, running, carrying heavy groceries or other loads upstairs, shoveling snow, or participating in a strenuous fitness class, and fast swimming
64–76% HRmax		77–93% HRmax	

*Absolute rates of energy expenditure during physical activity are commonly described as light, moderate, and vigorous intensity. Energy expenditure is expressed by multiples of the metabolic equivalent of task (MET), where one MET is the rate of energy expenditure while sitting at rest. This approach is not useful in reasonably healthy individuals because their boundary conditions will occur at higher METs. HRR, heart rate reserve (max HR-rest HR). HRmax, maximal heart rate, preferably measured during an incremental exercise test but otherwise estimated as 220-age. RPE, rating of perceived exertion on a 10 point scale. RPE and the verbal descriptions here are the most useful for exercise prescription, but an incremental test is the most accurate way to identify the appropriate boundary conditions and prescribe exercise*.

Moderate and vigorous physical activity is described in multiples of resting MET. Given that V̇O_2_max can be <5 METs to >20 METs, depending on age, sex, genetic predisposition, and individual fitness level, a MET-based recommendation would represent a wide range of disturbances to homeostasis (Iannetta et al., 2021). This problem is further complicated by the fact that individuals have no way of quantifying the MET value of their exercise.

Examples of moderate (e.g., brisk walking, dancing, and gardening) and vigorous intensity exercise (e.g., jogging, running, fast cycling, fast swimming, and walking briskly up a hill) are useful in describing exercise intensity for prescription. However, they provide no objective means for relating exercise intensity to the individual disturbance to homeostasis.

Prescription of moderate and vigorous exercise intensity may also be based on heart rate ranges expressed relative to an individual maximal heart rate or percentage of heart rate reserve, where moderate exercise is 40–59% of aerobic capacity reserve or heart rate reserve and vigorous is 60–84% of these reserves (Warburton et al., 2007). While these methods are certainly more appropriate than prescribing at a given absolute speed or power output, or even as multiples of resting MET, exercising at a common percentage of V̇O_2_max or heart rate reserve may represent different magnitudes of disturbance to homeostasis for different individuals (Iannetta et al., 2020a). For example, two individuals exercising at 70% of V̇O_2_max could be exercising in different exercise domains and therefore experiencing different levels of metabolic disturbance to homeostasis (Jamnick et al., 2020).

## Moderate and Vigorous Exercise Intensity

Current definitions and descriptions of moderate to vigorous intensity exercise are unclear. The lack of consistency and clarity of these definitions has several implications. First, given the multiple formats used for describing moderate and vigorous intensity exercise in the research literature, ***it is difficult to relate intensity to health outcomes. Second, this ambiguity also makes it challenging for practitioners prescribing exercise and their clients to fully understand what moderate to vigorous intensity exercise is***.

### Moderate and Vigorous Exercise Intensity Defined

There is not a clear boundary condition marking the lower limit of moderate exercise intensity. The upper limit is marked by VT1 or LT1. Moderate exercise intensity can be accomplished with negligible disturbance to homeostasis and could be maintained for hours. The VT1 and VT2 (or LT1 and LT2) serve as lower and upper boundaries, respectively, for vigorous intensity exercise. Vigorous exercise intensity can be sustained with entirely aerobic metabolism but will result in progressively more disturbance to homeostasis through this range of intensity. At VT2 or LT2, exercise duration is typically limited to 30–50min (Hoogeveen et al., 1997).

To encourage people to engage in the appropriate intensity of exercise and to guide future research concerned with the health benefits of exercise, it is important to have objective methods to quantify the intensity of exercise.

## Monitoring Tools To Target Moderate and Vigorous Intensity Domains

Heart rate provides immediate and objective feedback on the intensity of exercise, but individualized testing is recommended so appropriate HR ranges can be prescribed to coincide with an individual’s relative intensity domains. Given that individualized testing may not be accessible, rating of perceived exertion may be a practical alternative. The perceived effort to sustain a given exercise intensity coincides with the physiological changes or changes in homeostasis occurring to support the energy demand of the activity. The perception of effort appears to allow detection of disturbance to homeostasis consistent with moderate or vigorous intensity. A study conducted on 2,560 participants evaluating Borg’s 6–20 rating of perceived exertion scale determined a mean value of 10.8±1.8 at the boundary between moderate and vigorous and 13.6±1.8 for the upper limit of vigorous exercise (Scherr et al., 2013). Thus, rating of perceived exertion, which can be refined and individually scaled using an incremental test, may be a practical, effective method of identifying moderate to vigorous exercise. Periodic reassessment will provide motivational feedback and an opportunity to modify the exercise prescription.

An alternative to a subjective rating of perceived exertion is the talk-test (Reed and Pipe, 2014). This simple approach allows approximating the VT1 by awareness of the intensity of exercise, where ventilation becomes sufficient to make it somewhat difficult to carry on a conversation. Just below this intensity is considered moderate. Above this intensity, when conversation is challenging, the intensity is vigorous (Creemers et al., 2017). When conversation is impossible, the intensity of exercise is greater than vigorous.

## Conclusion

Although any physical activity is beneficial in lieu of a completely sedentary lifestyle, it is recommended that adults obtain 150min of moderate to vigorous intensity exercise or 75min of vigorous intensity exercise each week. Intensity of exercise relates to metabolic disturbance to homeostasis. Moderate intensity exercise should approach the VT1or LT1 and vigorous exercise is between the VT1 or LT1 and VT2 or LT2. Subjective perception of exercise can allow the detection of these intensities. Those conducting research on the health benefits of exercise and those prescribing exercise should become familiar with these expressions of intensity and use them appropriately.

## Author Contributions

BM, JM, DK, and JW contributed to extensive discussions and exchange and edits in the preparation of this perspective article. Each author contributed some original work and all contributed in the editing process. All authors contributed to the article and approved the submitted version.

## Funding

Funding for this work was provided by the Natural Science and Engineering Council of Canada (grant No 1032434).

## Conflict of Interest

The authors declare that the research was conducted in the absence of any commercial or financial relationships that could be construed as a potential conflict of interest.

## Publisher’s Note

All claims expressed in this article are solely those of the authors and do not necessarily represent those of their affiliated organizations, or those of the publisher, the editors and the reviewers. Any product that may be evaluated in this article, or claim that may be made by its manufacturer, is not guaranteed or endorsed by the publisher.

## Footnotes

^1^ https://health.gov/sites/default/files/2019-09/Physical_Activity_Guidelines_2nd_edition.pdf
